# A common phytoene synthase mutation underlies white petal varieties of the California poppy

**DOI:** 10.1038/s41598-019-48122-3

**Published:** 2019-08-12

**Authors:** Andrew J. Pollack, Xue Gong, Jonathan R. Pollack

**Affiliations:** Department of Pathology, Stanford University School of Medicine, Stanford, California USA

**Keywords:** Natural variation in plants, Plant genetics

## Abstract

The California poppy (*Eschscholzia californica*) is renowned for its brilliant golden-orange flowers, though white petal variants have been described. By whole-transcriptome sequencing, we have discovered in multiple white petal varieties a single deletion leading to altered splicing and C-terminal truncation of phytoene synthase (PSY), a key enzyme in carotenoid biosynthesis. Our findings underscore the diverse roles of phytoene synthase in shaping horticultural traits, and resolve a longstanding mystery of the regaled golden poppy.

## Introduction

The California poppy (*Eschscholzia californica*), also known as the golden poppy, is native to the West Coast of the United States^1,2^. The flowers are brilliant golden-orange, instantly recognizable, and widely drawn and photographed. Native Americans valued the golden poppy as a food source. First catalogued from a Russian seafaring expedition to the San Francisco Bay in the early1800s, the golden poppy was designated the state flower of California in 1903. The golden poppy has since been inextricably linked to California pop culture, even eulogized by the novelist John Steinbeck^2^.

**Figure 1:**
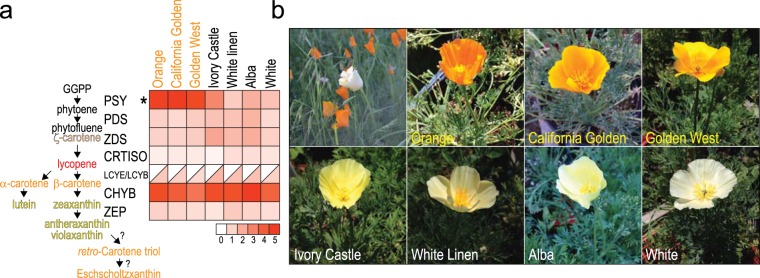
White-petal California poppy varieties show reduced flower PSY transcript. (**a**) *Left*, Carotenoid biosynthetic pathway, with pigment colors approximated by colored text. Abbreviations: GGPP, geranylgeranyl diphosphate; PSY, phytoene synthase; PDS, phytoene desaturase; ZDS, ζ-carotene desaturase; CRTISO, carotenoid isomerase; LCYE, lycopene ε-cyclase; LCYB, lycopene β-cyclase; CHYB, carotene β-hydroxylase; ZEP, zeaxanthin epoxidase. *Right*, heatmap depicts flower bud transcript levels of the major carotenoid biosynthetic pathway genes, normalized to housekeeping gene EIF4A2. Note, only phytoene synthase (PSY) transcript levels are significantly altered in white-petal varieties, which show on average 2.5-fold reduction (*P* = 0.003). (**b**) Representative California poppy varieties studied (clockwise from *top left*): single white poppy in a field of golden poppies; Orange; California Golden; Golden West; White; Alba; White Linen; and Ivory Castle.

The golden-orange color results from carotenoid pigments^3^. The carotenoid biosynthetic pathway in plants has been well characterized^4,5^. The first committed step is the condensation of two geranylgeranyl diphosphate (GGPP) molecules to phytoene (colorless), catalyzed by phytoene synthase (PSY) (Fig. 1a, *left*). Subsequent enzymatic steps that include desaturation, isomerization, cyclization, hydroxylation and epoxidation sequentially generate carotenoids that appear red (lycopene), orange (α-carotene and β-carotene), and yellow (lutein, zeaxanthin, antheraxanthin, and violaxanthin), and combinations of these pigments create the observed palette. Notably, California poppy petals also contain abundant retro-carotenoids (*retro*-carotene triol and Eschscholzxanthin), generated from antheraxanthin and violaxanthin by as yet unknown enzymes^6,7^. Additional proteins have been reported to modulate carotenoid biosynthesis or degradation^5,8^. Carotenoids serve not only as chromoplast pigments to attract pollinators and horticulturalists, but also as chloroplast accessory pigments and antioxidants crucial for photosynthesis^4^.

For those living in or visiting California, it is not uncommon to spot the occasional white-petal California poppy in a field of orange poppies. Indeed, white-petal varieties were described from English garden hybrids as early as the 1880s^9^, and in scientific literature from the 1930s^10^. Biochemical and genetic studies ensued, defining the white-petal trait to be recessive and based on near absence of carotenoid pigment^11^. By crossing different white-petal variants, including 8 originating from natural populations and 7 from commercial sources, Barrell *et al*.^12^ reported lack of complementation indicative of a single genetic locus. However, the gene and mutation(s) underlying white-petal variants have yet to be discovered.

## Results and Discussion

To investigate the genetic basis of white-petal poppy variants, we carried out transcriptome sequencing (RNAseq) of developing flower buds from four different commercial white poppy varieties displaying varied shades of white: Ivory Castle, White Linen, Alba, and White (Fig. 1b). Three orange-petal poppy varieties (Orange, California Golden, and Golden West) served as controls. RNA was isolated from developing flower buds, where pigment production was presumed ongoing. Since no poppy reference genome was available, RNAseq reads were assembled *de novo* into transcript contigs, which were then annotated by homology to an orthologous reference transcriptome, for which we selected another eudicot clade flowering plant, the garden tomato (*Solanum lycopersicum*).

Since white poppy petals are deficient in carotenoid pigments^11^, we focused on genes of the carotenoid biosynthetic pathway. Comparing expression of carotenoid biosynthesis genes between white and orange poppy varieties, only phytoene synthase (*PSY*) showed significantly altered expression, with an average 2.5-fold reduced transcript levels in white varieties (*P* = 0.003, two-sided Student’s t-test) (Fig. 1a, *right*). While this finding focused attention on *PSY*, the modest reduction in white varieties was unlikely to account for a near absence of carotenoid pigment.

**Figure 2:**
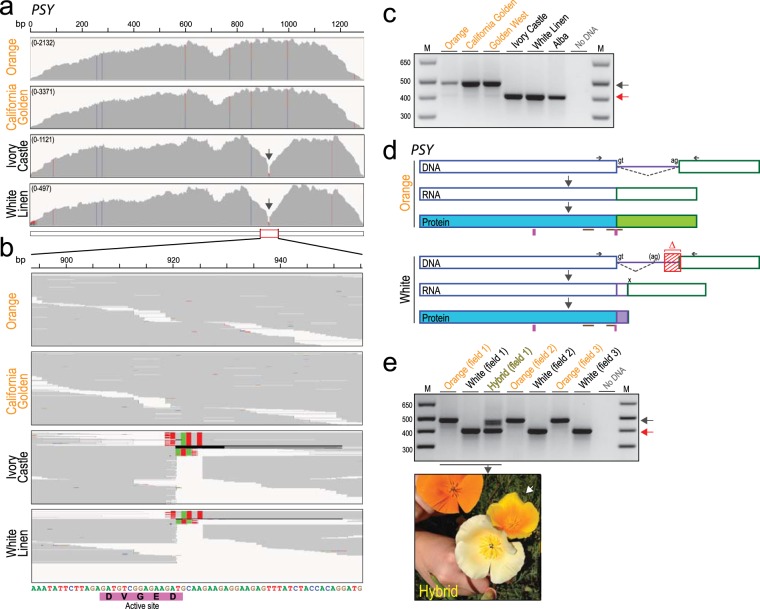
White-petal California poppy varieties harbor a frameshifting deletion in *PSY*. (**a**) Integrative Genomics Viewer (IGV) coverage plots and alignments for RNAseq reads spanning the *PSY* coding sequencing, shown each for two representative orange-petal (Orange and California Golden) and white-petal (Ivory Castle and White Linen) varieties. Mismatches (polymorphisms or mutations) relative to the reference (Orange) are indicated by color-coded bars. Note the alignment gap in the two white-petal varieties (black arrow). (**b**) Close-up view highlighting the 5 bp alignment gap in the white-petal varieties. Note, the alignment gap overlaps with a putative enzyme active site (DXXXD motif, indicated below). (**c**) PCR across the exon-exon junction (site of alignment gap) results in a shorter PCR product (red arrow), indicative of genomic DNA deletion in the white-petal varieties. Image of full-length gel is available in Supplementary Fig. 4. (**d**) Illustration summarizing the *PSY* gene, mRNA and protein products inferred from sequence alignment of the PCR products (PCR primers indicated; see Fig. 3 for annotated cDNA sequences). In all four white-petal varieties, a 76 bp intronic deletion (red hatched box) ablates the 3′ splice acceptor. Usage of a cryptic splice acceptor leads to a coding frameshift with early translational termination. The resulting C-terminal truncation destroys one of two highly-conserved putative enzyme active sites (pink rectangles); brown lines indicate PSY conserved motifs. (**e**) PCR analysis (and subsequent sequencing) reveals the identical 76 bp intronic deletion in three white-petal poppy plants discovered among ostensibly wild California poppy fields from three geographically distinct locales. Note, a yellow-orange petal poppy plant discovered in Field 1 (also pictured in inset, white arrow) carries both the *PSY* wildtype and deletion allele, suggesting that it represents an F1 hybrid cross between an orange and white petal variety. Image of full-length gel is available in Supplementary Fig. 4.

**Figure 3:**
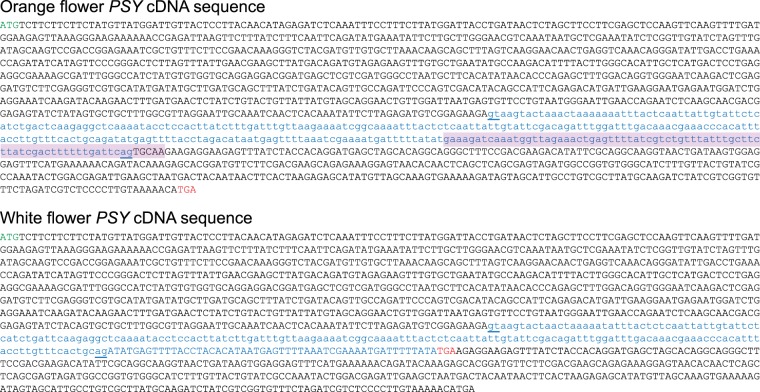
Poppy *PSY1* cDNA sequences. Shown are the flower bud *PSY* cDNA sequences (plus one intron) from Orange-petal (*top*) and White-petal (*bottom*) varieties, as determined from the RNAseq alignments and exon-spanning PCR. The intron sequences are in lower case text, with splice donor and acceptor sites underlined. The 76 bp sequence deleted in white varieties is indicated in the orange *PSY* cDNA by purple highlighting. The early termination codon (TGA) in the white *PSY* cDNA is indicated by red text.

Comparing the aligned *PSY* transcript reads between white and orange petal poppy varieties, all four white varieties (but none of the three orange varieties) exhibited an apparent 5 bp gap within the *PSY* transcript (Fig. 2a,b and Supplementary Fig. 1). The gap occurred within the coding region, at the site of an inferred exon-exon junction (by comparison to the tomato reference genome). To define the alteration at the genome level, we designed PCR primers to amplify and sequence across the exon-exon junction from genomic DNA (isolated from poppy seeds). In the white petal varieties, the resultant PCR product was consistently smaller (Fig. 2c). Sequence alignment revealed a 76 bp deletion within the *PSY* intron, which extended through the 3′ splice acceptor site and 5 bp of the downstream exon (explaining the apparent 5 bp alignment gap from the RNAseq reads) (Figs 2d, 3). By comparing the white-petal *PSY* genome sequence and assembled transcript contig, loss of the splice acceptor site led to usage of a cryptic splice acceptor site within the intron, resulting in a coding frameshift with early translational termination and predicted C-terminal truncation of the PSY protein (Fig. 2d and Fig. 3). Early translational termination is associated with nonsense-mediated mRNA decay^13^, consistent with our observed reduced *PSY* transcript levels. Notably, the C-terminal truncation abolishes a highly-conserved putative enzyme active site (DXXXD motif) in PSY (Fig. 2b,d)^14^.

That the *PSY* deletion is present in all four white petal varieties (minimally 8 alleles if diploid), but in none of the three orange petal varieties (minimally 6 alleles), demonstrates strong segregation with the white petal phenotype (*P* = 0.0003, two-sided Fisher’s exact test). Together, the genetic and inferred biochemical (predicted loss of active site) data provide strong evidence that the 76 bp deletion underlies the white petal trait. Moreover, that all four white petal poppy varieties (notwithstanding subtle differences in petal hues) harbor precisely the same deletion suggests that they were likely all derived from a single white-petal poppy origin.

In addition to studying commercial varieties, we also sought to examine white-petal specimens among wild poppy plants. To this end, we undertook expeditions to find and collect white petal specimens among California poppy fields across Santa Clara, San Mateo, and Solano counties. Of three specimens collected, all harbored precisely the same *PSY* mutation (Fig. 2e), suggesting that they likely represent commercial seed contaminants among orange petal varieties that were seeded rather than wild plants. Interestingly, in one field we noted orange and white poppies together with an uncommon yellow-orange petal variant. PCR analysis revealed that the yellow-orange poppy carried both the wildtype and deletion *PSY* allele, suggesting an F1 hybrid between previously seeded orange and white petal varieties (Fig. 2e).

**Figure 4:**
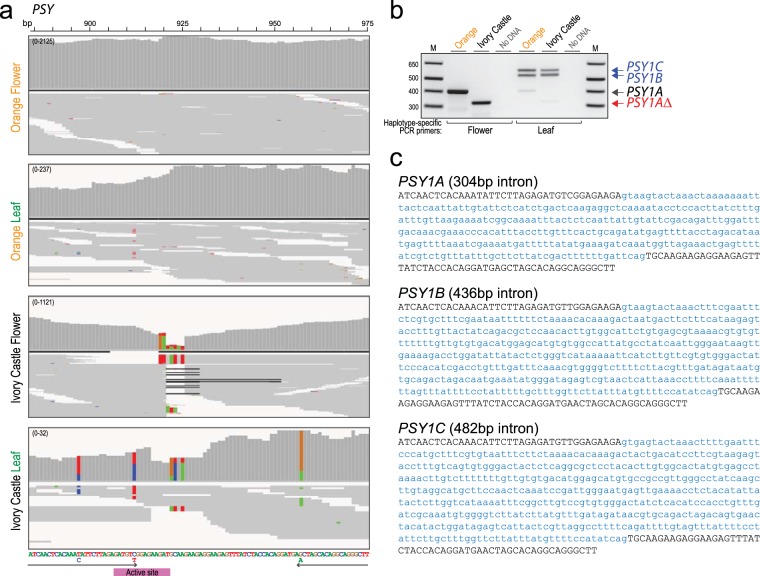
Poppy leaves express additional *PSY* genes. (**a**) IGV coverage plots and alignments for RNAseq reads spanning a portion of the PSY coding sequencing (bp 885–975), shown for Orange flower, Orange leaf, Ivory Castle flower, and Ivory Castle leaf. Note that the 5 bp alignment gap present in Ivory Castle flower is observed in only a minority of reads from Ivory Castle leaf. Note also in Ivory Castle leaf the presence of 3 SNPs flanking the gap that segregate with the non-deletion reads, permitting design of haplotype-specific (leaf *vs*. flower expressed) PCR primers (shown below). The 3 SNPs are also present in Orange leaf, but masked by the higher (flower) PSY expression. (**b**) Haplotype-specific PCR of genomic DNA across the exon-exon junction (site of alignment gap) using leaf-expression specific PCR primers results in two longer PCR products (blue arrows), distinct from the shorter flower-expression specific PCR products, and indicative of additional PSY genes (annotated *PSY1B* and *PSY1C*) expressed in poppy leaves. Image of full-length gel is available in Supplementary Fig. 4. (**c**) Partial genome sequences for *PSY1A* (*top*), *PSY1B* (*middle*), and *PSY1C* (*bottom*), as determined by exon-spanning PCR. Intron sequences are in lower case text.

Carotenoids are flower petal pigments, but they also provide essential roles as accessory pigments and antioxidants in chloroplasts for photosynthesis^4^. Thus, given the *PSY* null mutation identified from flower buds, the existence of other PSY encoding genes seemed likely. To investigate that possibility, we carried out RNAseq from green leaf material from orange and white petal poppy varieties. Aligning the reads, only a small fraction of the PSY reads from the white-petal leaf specimen exhibited the deletion (Fig. 4a). A distinct set of single nucleotide polymorphisms (SNPs) present only in the leaf RNA segregated with the non-deletion reads, allowing us to design haplotype-specific PCR primers to amplify across the exon-exon junction. Notably, PCR of genomic DNA using the non-deletion haplotype-specific primers revealed two larger PCR products (Fig. 4b), where sequencing disclosed two different intron sequences (Fig. 4c). This finding indicates the presence of two additional *PSY* genes (which we have designated *PSY1B* and *PSY1C*), expressed in poppy leaves.

Based on the relative frequencies of SNPs in the PSY transcripts from petal and leaf tissue (Supplementary Fig. 2 and Supplementary Table 1), we can infer that *PSY1A* (harboring the mutation in white petal varieties) is the only *PSY* gene expressed in California poppy petals, while *PSY1B* and *PSY1C* (indistinguishable from one another by SNPs) are expressed only in leaves. Nonetheless, *PSY1A* is also expressed in leaves where indeed it is more abundantly expressed (accounting for 97% of leaf *PSY* transcripts in orange petal varieties, reduced to 78% in white-petal varieties) compared to *PSY1B*/*1C*. California poppy *PSY1A* exhibits high (99%) homology to *PSY1B/1C* at the nucleotide sequence level, and 100% identity at the amino acid sequence level, suggesting relatedness by recent gene duplication. However, we note limitations of our analysis, including variable read coverages (particular at the ends of the *PSY* genes), and the challenges of phasing short reads and assigning SNPs to individual genes. A definitive analysis will require cloning the individual *PSY* cDNAs and genome loci.

**Figure 5:**
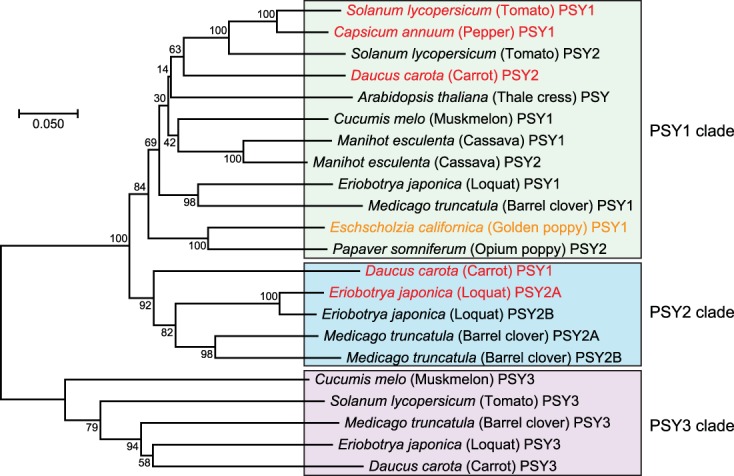
Phylogenetic analysis of PSY protein sequences. Phylogram depicts phylogenic relationship of California poppy PSY and 21 other eudicot PSY proteins. Branch lengths are in units of the number of amino acid substitutions per site. Bootstrap values (percentage of trees in which the associated taxa clustered together in 500 re-samplings) are indicated next to the branches. Clades are based on the Stauder *et al*. designation^20^. *PSY* genes specifically expressed in single carotenoid-rich organs are indicated by red text. Note, *PSY* gene nomenclature varies considerably by species.

The finding of multiple *PSY* gene paralogs in plants, first detailed in the tomato^15^, is now common. Like for the California poppy, some such *PSY* paralogs are expressed primarily in green (photosynthetic) tissues, while others drive carotenoid accumulation in flowers, fruits, or roots. For example, in the tomato (*Solanum lycopersicum*), *PSY1* is predominantly expressed in the petals and ripening fruit, while *PSY2* is predominant in leaves^16^. In the loquat (*Eriobotrya japonica*), *PSY1* is expressed in the fruit peel, *PSY2A* in the ripening fruit flesh, and *PSY2B* in leaves^17^. And in the carrot (*Daucus carota*), *PSY1* and *PSY2* are expressed in the root, while *PSY1* is also expressed in leaves^18^. A comparative analysis of PSY protein sequences among eudicots reveals California poppy PSY to be most closely related to PSY from the recently sequenced opium poppy (*Papaver somniferum*) genome^19^, and overall more closely related to the so-called Eudicot PSY1 clade (Fig. 5 and Supplementary Fig. 3)^20^.

In summary, by transcriptome sequencing of California poppy flower buds, we have identified a frameshifting deletion in phytoene synthase that is common to multiple commercial white petal varieties. All have distinct white hues (likely due to different genetic backgrounds), but nonetheless appear to have been bred from the same common originator. Importantly, the white-petal trait in 15 different natural and commercial California poppy variants was previously shown to map to a single genetic locus^12^. That study included the Alba and Ivory Castle varieties also analyzed here. Thus, we can infer that *PSY1A* mutations underlie all previously studied white-petal California poppy variants. Whether other variants share the same 76 bp frameshifting deletion mutation remains to be determined.

PSY variants/mutations have previously been associated with agriculturally important traits, e.g., color variation in tomatoes, peppers, cassavas, and loquats^17,21–23^. We have now also connected *PSY* mutations to ornamental horticulture. Our discovery resolves a decades old mystery of the molecular underpinnings of white-petal California poppies, and adds to the cultural legacy of the California golden poppy.

## Methods

### Plant materials

Commercial California poppy varieties were purchased as seeds from Eden Brothers (Ivory Castle, White Linen, Alba, Orange, and Golden West), Vermont Wildflowers (White), and Cornucopia (California Golden). Seeds were germinated in individual pots, and subsequent developing flower buds collected and frozen on dry ice. In some cases, poppy leaf material was also collected. Mature flowers from the same plants were examined and photographed to verify the advertised varieties. We also collected ostensibly wild California poppy flower samples from fields across three San Francisco Bay Area counties. For RNA isolation, plant material (flower buds with calyx caps removed, or leaves) was pulverized in liquid nitrogen using a mortar and pestle, and then RNA prepared using the RNeasy Mini kit (Qiagen). Genomic DNA was isolated from commercial seeds, using the Quick-DNA Plant/Seed Miniprep Kit (Zymo Research).

### Transcriptome sequencing

For transcriptome sequencing, RNAseq libraries were generated from 1 µg RNA using Illumina TruSeq RNA Library Prep Kit v2, and barcoded libraries sequenced (101 bp × 2 for flower buds, 50 bp × 1 for leaves) on an Illumina HiSeq 2000 to an average depth of 27 million reads per sample. Reads were then assembled de novo into transcript contigs using Trinity^24^, implemented within FRAMA^25^, using the garden tomato (*Solanum lycopersicum*) transcriptome^26^ (Assembly SL2.50, accessed from EnsemblePlants) as an orthologous reference to assign gene annotations. Annotated transcripts were quantified as Reads Per Kilobase of transcript per Million mapped reads (RPKMs). Reported transcript levels for carotenoid biosynthetic pathway genes were normalized to the housekeeping gene *EIF4A2*. Aligned reads were visualized against the Orange (Eden Brothers) variety, using Integrative Genomics Viewer (IGV)^27^.

### PCR analysis

PCR was done using AmpliTaq Gold polymerase and reagents (Applied Biosystems), with 100 ng input DNA and 40 cycles (94 °C 30 s, 54 °C 30 s, 72 °C 60 s). PCR/sequencing primers were PSY-Gap-F 5′-TCAAGCAACGACGGAGAGTA; PSY-Gap-R 5′-CCTTGCCTGCGAATATGTCT; PSY-Flower-F 5c-AAATCAACTCACAAATATTCTTAGAGATGTC; PSY-Flower-R 5′-GCCCTGCCTGTGCTAGC; PSY-Leaf-F 5′-ATCAACTCACAAACATTCTTAGAGATGTT; PSY-Leaf-R 5′-AAGCCCTGCCTGTGCTAGT. PCR products were purified with the QIAquick PCR Purification kit (Qiagen), and then Sanger-sequenced (Quintara Biosciences). Sequence reads were aligned using NCBI BLAST Align two sequences tool.

### Phylogenetic analysis

Multiple sequence alignment of PSY proteins was done using Clustal Omega^28^, using default parameters and the following protein accessions: *Arabidopsis thaliana* PSY (AAA32836.1); *Capsicum annuum* (Pepper) PSY1 (ACE78189.1); *Cucumis melo* (Muskmelon) PSY1 (AEH03200.1), PSY3 (formerly PSY2) (AEH03199.1); *Daucus carota* (Carrot) PSY1 (ABB52067.1), PSY2 (ABB52068.1), PSY3 (XP_017217851.1); *Eriobotrya japonica* (Loquat) PSY1 (AIT18246.1), PSY2A (AIT18247.1), PSY2B (AIT18249.1), PSY3 (AIT18250.1); *Manihot esculenta* (Cassava) PSY1 (ACY42666.1), PSY2 (ACY42670.1); *Medicago truncatula* (Barrel clover) PSY1 (AES99105.1), PSY2A (KEH33671.1), PSY2B (AET00322.2), PSY3 (AES71870.1); *Papaver somniferum* (Opium poppy) PSY2 (XP_026387400.1); *Solanum lycopersicum* (Tomato) PSY1 (P08196.2), PSY2 (ABV68559.1), PSY3 (XP_004228928.1). Phylograms were constructed with MEGA X^29^, using the Neighbor-Joining method with default parameters.

### Accession codes

RNAseq data are available through the NCBI Short Read Archive (accession PRJNA517727). PSY sequences are available through GenBank (accessions MK620867-MK620871).

## Supplementary information

Supplementary Tables and Figures
