# Supplementary material for: A common phytoene synthase mutation underlies white petal varieties of the California poppy
Source: Sci Rep. 2019 Aug 12;9:11615. doi: 10.1038/s41598-019-48122-3 (PMC6690985; doi:10.1038/s41598-019-48122-3)
Supplement: Supplementary file 1 — Supplementary Tables and Figures [file 41598_2019_48122_MOESM1_ESM.pdf]

A common phytoene synthase mutation underlies white petal varieties of the California poppy

Andrew J. Pollack[1], Xue Gong[1], Jonathan R. Pollack[1,*]

[1]Department of Pathology, Stanford University School of Medicine, Stanford, California, USA

Supplementary Tables and Figures

Supplementary Table 1: *PSY* SNP frequencies in leaves of orange and white petal varieties

SNP #	Nucleotide Position	Major allele	Minor allele	White-petal leaf #1, Minor allele frequency	White-petal leaf #2, Minor allele frequency	Orange-petal leaf, Minor allele frequency
1	528	T	C	0[a]	13	0
2	534	C	T	0[a]	18	2
3	546	T	G	14	56	2
4	558	T	C	14	36	7
5	582	G	A	17	28	7
6	690	A	G	0[a]	13	4
7	759	G	T	31	17	2
8	894	T	C	27	17	5
9	909	C	T	41	36	10
10	954	G	A	20	24	3
11	1098	G	T	13	1	1
12	1123	T	C	17	22	2
13	1131	G	A	21	23	2
14	1149	C	T	11	16	1
				Mean = 21.5	Mean = 22.9	Mean = 3.4

[a]Low coverage (<25 total reads); excluded from analysis

Fig. S1. White-petal California poppy varieties harbor a frameshifting deletion in *PSY*. Integrative Genomics Viewer (IGV) coverage plots and alignments for RNAseq reads spanning the *PSY* coding sequencing, shown each for three orange-petal (Orange, California Golden, Golden West) and four white-petal (Ivory Castle, White Linen, Alba and White) varieties. Mismatches (polymorphisms or mutations) relative to the reference (Orange) are indicated by color-coded bars. Note the alignment gap in the four white-petal varieties (black arrow).

Fig. S2. California poppy *PSY1A* versus *PSY1B*/*1C* cDNA sequences. Shown are the *PSY1A* (top) and *PSY1B*/*1C* (bottom) cDNA sequences inferred from short-read RNAseq. The 14 identified SNPs specific to the *PSY1B*/*1C* transcripts are indicated in red text.

Fig. S3. Multiple sequence alignment of eudicot PSY proteins. Sequence alignment performed using Clustal Omega. Conservation indicated: full (*), strong (:), weak (.). Enzyme active sites (DXXXD motif) highlighted in blue.

Fig. S4. Full-length gels. Shown are the full-length gels corresponding to the cropped images presented in Figs. 2c, 2e, and 4b. Molecular weight markers are indicated.

Figure S1

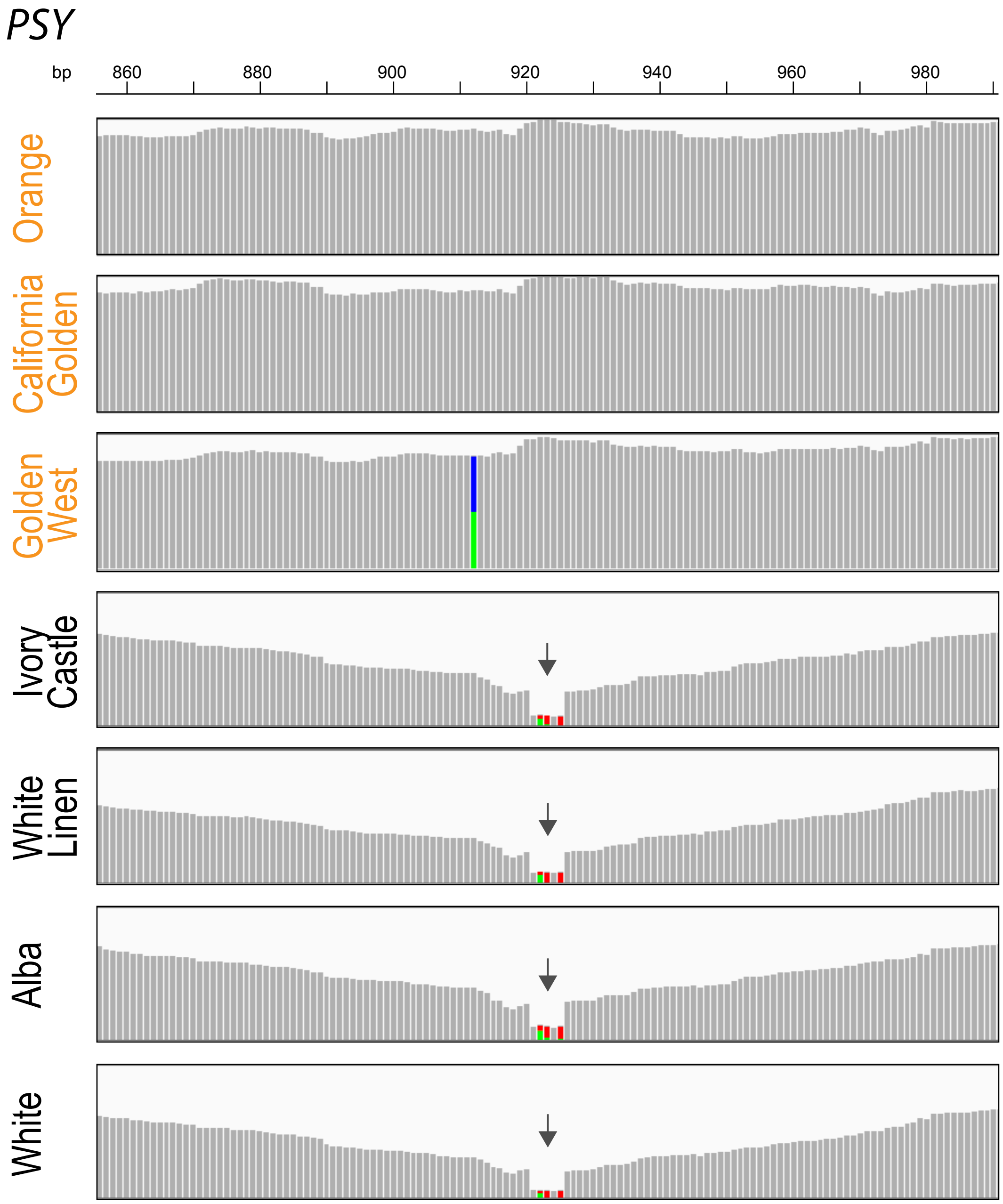

PSY
bp
860
880
900
920
940
960
980
Orange
California Golden
Golden West
Ivory Castle
White Linen
Alba
White

Figure S2

>Eschscholzia californica PSY1A
ATGTCTTCTTCTTCTATGTTATGGATTGTTACTCCTTACAACATAGAGATCTCAAATTTCCTTTCTTATGGATTACCTGATAA
CTCTAGCTTCCTTCGAGCTCCAAGTTCAAGTTTTGATGGAAGAGTTAAAGGGAAGAAAAAACCGAGATTAAGTTCTTTATCTT
TCAATTCAGATATGAAATATTCTTGCTTGGGAACGTCAAATAATGCTCGAAATATCTCGGTTGTATCTAGTTTGATAGCAAGT
CCGACCGGAGAAATCGCTGTTTCTTCCGAACAAAGGGTCTACGATGTTGTGCTTAAACAAGCAGCTTTAGTCAAGGAACAACT
GAGGTCAAACAGGGATATTGACCTGAAACCAGATATCATAGTTCCCGGGACTCTTAGTTTATTGAACGAAGCTTATGACAGAT
GTAGAGAAGTTTGTGCTGAATATGCCAAGACATTTTACTTGGGCACATTGCTCATGACTCCTGAGAGGCGAAAAGCGATTTGG
GCCATCTATGTGTGGTGCAGGAGGACGGATGAGCTCGTCGATGGGCCTAATGCTTCACATATAACACCCAGAGCTTTGGACAG
GTGGGAATCAAGACTCGAGGATGTCTTCGAGGGTCGTGCATATGATATGCTTGATGCAGCTTTATCTGATACAGTTGCCAGAT
TCCCAGTCGACATACAGCCATTCAGAGACATGATTGAAGGAATGAGAATGGATCTGAGGAAATCAAGATACAAGAACTTTGAT
GAACTCTATCTGTACTGTTATTATGTAGCAGGAACTGTTGGATTAATGAGTGTTCCTGTAATGGGAATTGAACCAGAATCTCA
AGCAACGACGGAGAGTATCTATAGTGCTGCTTTGGCGTTAGGAATTGCAAATCAACTCACAAATATTCTTAGAGATGTCGGAG
AAGATGCAAGAAGAGGAAGAGTTTATCTACCACAGGATGAGCTAGCACAGGCAGGGCTTTCCGACGAAGACATATTCGCAGGC
AAGGTAACTGATAAGTGGAGGAGTTTCATGAAAAAACAGATACAAAGAGCACGGATGTTCTTCGACGAAGCAGAGAAAGGAGT
AACACAACTCAGCTCAGCGAGTAGATGGCCGGTGTGGGCATCTTTGTTACTGTATCGCCAAATACTGGACGAGATTGAAGCTA
ATGACTACAATAACTTCACTAAGAGAGCATATGTTAGCAAAGTGAAAAAGATAGTAGCATTGCCTGTCGCTTATGCAAGATCT
ATCGTCGGTGTTTCTAGATCGTCTCCCCTTGTAAAAACATGA

>Eschscholzia californica PSY1B/1C
ATGTCTTCTTCTTCTATGTTATGGATTGTTACTCCTTACAACATAGAGATCTCAAATTTCCTTTCTTATGGATTACCTGATAA
CTCTAGCTTCCTTCGAGCTCCAAGTTCAAGTTTTGATGGAAGAGTTAAAGGGAAGAAAAAACCGAGATTAAGTTCTTTATCTT
TCAATTCAGATATGAAATATTCTTGCTTGGGAACGTCAAATAATGCTCGAAATATCTCGGTTGTATCTAGTTTGATAGCAAGT
CCGACCGGAGAAATCGCTGTTTCTTCCGAACAAAGGGTCTACGATGTTGTGCTTAAACAAGCAGCTTTAGTCAAGGAACAACT
GAGGTCAAACAGGGATATTGACCTGAAACCAGATATCATAGTTCCCGGGACTCTTAGTTTATTGAACGAAGCTTATGACAGAT
GTAGAGAAGTTTGTGCTGAATATGCCAAGACATTTTACTTGGGCACATTGCTCATGACTCCTGAGAGGCGAAAAGCGATTTGG
GCCATCTATGTGTGGTGCAGGAGGACGGAcGAGCTtGTCGATGGGCCgAATGCTTCACAcATAACACCCAGAGCTTTGGACAG
aTGGGAATCAAGACTCGAGGATGTCTTCGAGGGTCGTGCATATGATATGCTTGATGCAGCTTTATCTGATACAGTTGCCAGAT
TCCCAGTCGACATACAGCCATTCAGgGACATGATTGAAGGAATGAGAATGGATCTGAGGAAATCAAGATACAAGAACTTTGAT
GAACTCTATCTtTACTGTTATTATGTAGCAGGAACTGTTGGATTAATGAGTGTTCCTGTAATGGGAATTGAACCAGAATCTCA
AGCAACGACGGAGAGTATCTATAGTGCTGCTTTGGCGTTAGGAATTGCAAATCAACTCACAAAcATTCTTAGAGATGTtGGAG
AAGATGCAAGAAGAGGAAGAGTTTATCTACCACAGGATGAaCTAGCACAGGCAGGGCTTTCCGACGAAGACATATTCGCAGGC
AAGGTAACTGATAAGTGGAGGAGTTTCATGAAAAAACAGATACAAAGAGCACGGATGTTCTTCGACGAAGCAGAGAAAGGAGT
AACACAACTCAGCTCAGCtAGTAGATGGCCGGTGTGGGCATCTcTGTTACTaTATCGCCAAATACTGGAtGAGATTGAAGCTA
ATGACTACAATAACTTCACTAAGAGAGCATATGTTAGCAAAGTGAAAAAGATAGTAGCATTGCCTGTCGCTTATGCAAGATCT
ATCGTCGGTGTTTCTAGATCGTCTCCCCTTGTAAAAACATGA

Figure S3

```
Arabidopsis thaliana (Thale cress) PSY        MSSSVAVLWVATSS-LNPDPMNNCGLV-------RVLESSRLFSPCQNQRLNKG--KKKQIPTWSSSFVRN-------------RSRRIGVVSSSLVASP-SGEIAL---SSEEKVYNVV  93
Eschscholzia californica (Golden poppy) PSY1 -MSSSSMLWIVTPYNIEISNFLSYGL----------PDNSSFLRA-PSSSFDGRVKGKKK-PRLSSLSFNSDMKYSCLGT--SNNARNI-SVVSSLIASP-TGEIAV---SSEQRVYDVV  100
Papaver somniferum (Opium poppy) PSY2        --MSCALLY-VVSPNTEISKFISYGL----------PDYSI-KAA-LSSNFDGRIKGKKKGTRWSSFSFTSDLKYTCLSGGGSDNAGTF-PVVSSLVANS-AAEVAV---SSEQRVYDVV  100
Medicago truncatula (Barrel clover) PSY1     --MSVTLLRVASSPSLEV-SC-SNGLLDSFG-VVKLVDSSKFLSRDFVSIR--AKK-NDKKKTRRFCSLSTDIKYACVGQPGLESASNF-PLLSNVLANPTAGEVTV---SSEQKVYDVV  108
Cucumis melo (Muskmelon) PSY1                --MSLASSLVVSS-NVELSPS-SFGFLDSVRDGPQIPDSFRFSSRNRVPN-----L-INKKQKWGNHSHSTELKYPILHESGYGS-----VIVASMVANP-AGEIAV---SAEQKVYNVV  101
Eriobotrya japonica (Loquat) PSY1            --MSVALIWVVSA-NTEV-FK-FYGILDSSR----------FVLGNR---SSIRAK-MGAKQDWKSCSLSTHVKYSSVGGSGLGSETKF-PVLLSMVANP-LGESAV---SSEQKVYDVV  96
Manihot esculenta (Cassava) PSY1             --MTIALLWVAIP-STEV-SN-SFGFFHSV----GALDSAKFGSVDRSLMFKKRAK-KGMNQKWKSSTVNVDLTNPCIG---LGSGSNL-PVISSMVASS-AGEMAV---SSEEKVYNVV  102
Manihot esculenta (Cassava) PSY2             --MTVALLWVAIP-STEV-SN-SFGFFHSV----RVLDSSKFGCVDRNLMFKGKAK-KGRNQKWKSGSVSIDLRSTCIG-----SGRKL-PIISSMVASH-AGEIAI---SSEEKVYNVV  100
Daucus carota (Carrot) PSY2                  --MSVAMSWIVTP-SLEV-SN-CFGYLETAREGTRVLDPSRLGSRDKNMRCGGRLE-KGKLRKWSSKSFNAEYSYSCLGGSELENGSIF-PVHSSMVVSA-DGDMAV---SSEKKVYDVV  109
Solanum lycopersicum (Tomato) PSY2           --MSVALLWVVSP-NSEV-SY-GTGFLDSVREGNRGLESSRFPSRDRNSMWKGGFK-KGGRQGWNFGFLNADLRYSCLGRSRTENGRSF-SVQSSLVASP-AGEMAV---SSEKKVYEVV  109
Solanum lycopersicum (Tomato) PSY1           --MSVALLWVVSP-C-DV-SN-GTSFMESVREGNRFFDSSRHRNLVSNERI---NR-GGGK-------------------QTNNGRKF-SVRSAILATP-SGERTM---TSEQMVYDVV  85
Capsicum annuum (Pepper) PSY1                --MSVALLWVVSP-C-DV-SN-GTGFLVSVREGNRIFDSSGRRNLACNERI---KR-GGGKQRWSFGS--------YLGGAQTGSGRKF-SVRSAIVATP-AGEMTM---SSERMVYDVV  97
Daucus carota (Carrot) PSY1                  ---------MACNFAVRVIYY-PKEIHGVS--------------VLNTNR---------S--------RKSRFSCRVMK---LS----TGVSAV-----AANPVR---TSEERVYEVV  62
Eriobotrya japonica (Loquat) PSY2A           --MSVVLLWVVS-PKQNAS-----SLLGLM--------------PRICT----------P--------RRSKF-CPKLG---FSSRV-LAYSGA-----VVNPAR---SSEEKVYEVV  65
Eriobotrya japonica (Loquat) PSY2B           --MSGVLLWVVS-PKENAN-----SLLGLL--------------PRICT----------P--------RRSKL-CSKLG---FSSGV-LAYSGA-----VANPAR---SSEEKVYEVV  65
Medicago truncatula (Barrel clover) PSY2A    --MSGVLLWVNCGLKENAI-----SLMGLV--------------GRDVRT---------Q--------RRFRH-CSGIS---FA-----CFSSA-----VVEPTR---SSEERVYEVV  63
Medicago truncatula (Barrel clover) PSY2B    --MSSVVVRVNCDAKS---------FVGI---------------SRIGRR---------E--------RSVRL-CSKVR---FG-----SGGAV-----VAEPSR---SSEERVYEVV  58
Solanum lycopersicum (Tomato) PSY3           --MCPATLSYSSNSWLN----------------------------ARNEIFSSKKCKTK-------LLI---------------------RVDQVLTVSPKKSEQSIHELLSVQGISHTH  62
Cucumis melo (Muskmelon) PSY3                --MSSCV-------FVT----------------------------PKSLT-FIGECKGNVFPKRFKTI----------------------RNGGGIIAAPRSSETLKLQTLSKQGIPLDH  60
Daucus carota (Carrot) PSY3                  --MSSTY-------YVA----------------------------PKVC--LRDQSNGKVVTFWNG------------------------IRAEATVTVPTRKIATKFTELSVQSIPHSD  57
Eriobotrya japonica (Loquat) PSY3            --MCSTI-------SFA----------------------------GKTY--IGE-SNGRI-RRRKSMVT---------------------AAKAQVITAPKQRSRPVFPELSIQGIPLAD  58
Medicago truncatula (Barrel clover) PSY3     --MSIPF-------SVA----------------------------AKPS--IIR-TNRKPYCRRYDV------------------------ITSELIMVPK-HKTATFPQLSKQGVSVAD  55
                                                                                                                                      : . :

Arabidopsis thaliana (Thale cress) PSY        LKQAALVNKQLRSSSYDLDVKKP--------QDVVLPG--SLSLLVGEAYDRCGEVCAEYPKTFYLGTLLMTPERRKAIWAIYVWCRRTDELVDGPNASHITPMALDRWEARLEDLFRGR  203
Eschscholzia californica (Golden poppy) PSY1 LKQAALVKEQLRSNR-DI--DLK--------PDIIVPG--TLSLL-NEAYDRCREVCAEYAKTFYLGTLLMTPERRKAIWAIYVWCRRTDELVDGPNASHITPRALDRWESRLEDVFEGR  206
Papaver somniferum (Opium poppy) PSY2        LKQAALVKEQLGSSG-QI--DVK--------PDIVVPG--TLSLL-SEAYDRCGEVCAEYAKTFYLGTLLMTPEKRKAIWAIYVWCRRTDELVDGPNASHITPKALDRWESRLEDLYAGR  206
Medicago truncatula (Barrel clover) PSY1     LKQASLVKRKLSSTG-EL--EVK--------PEIVLPG--NLSLL-SEAYDRCGEICAEYAKTFYLGTLLMTPERRRAIWAIYVWCRRTDELVDGPNASHITATAMDRWESRLDELFQGR  214
Cucumis melo (Muskmelon) PSY1                MKQAALVKRQLRTAG-EL--DVK--------PDIVLPG--TLSLL-NEAYDRCGEVCAEYAKTFYLGTMLMTPERQKAIWAIYVWCRRTDELVDGPNASHITPTALDRWEARLEELFQGR  207
Eriobotrya japonica (Loquat) PSY1            LKQASLGKKQLSSNG-YL--DVK--------RDIILPG--NLSLL-SKAYDRCGEVCAEYAKTFYLGTLLMTPERRRAIWAIYVWCRRTDELVDGPNASHITPTALDRWESRLDDLFQGR  202
Manihot esculenta (Cassava) PSY1             LKQAALVKKQLRSSE-NL--DAK--------TDIAVPG--TSSLL-SEAYDRCGEVCAEYAKTFYLGTLLMTPERRRAIWAIYVWCRRTDELVDGPNASHITPTALDRWEARLDDVFQGR  208
Manihot esculenta (Cassava) PSY2             LKQAALVKQQLKSSE-DL--DVK--------PDIVLPG--TLSLL-SEAYDRCGEVCAEYAKTFYLGTLLMTPERRRAIWAIYVWCRRTDELVDGPNASHITPTDLDRWEARLEDMFRGR  206
Daucus carota (Carrot) PSY2                  LKQAALVKRQFRSDE-EL--EVK--------PEMILPG--TLSLL-SEAYDRCGEVCAEYAKTFYLGTLLMTPERRRAIWAIYVWCRRTDELVDGPNASHITPSALDRWELRLEDLFKGR  215
Solanum lycopersicum (Tomato) PSY2           LKQAALVKRHLISTD-DI--QVK--------PDIVLPG--NLGLL-SEAYDRCGEVCAEYAKTFYLGTMLMTPDRRRAIWAIYVWCRRTDELVDGPNASHITPQALDRWEARLEDIFNGR  215
Solanum lycopersicum (Tomato) PSY1           LRQAALVKRQLRSTN-EL--EVK--------PDIPIPG--NLGLL-SEAYDRCGEVCAEYAKTFNLGTMLMTPERRRAIWAIYVWCRRTDELVDGPNASYITPAALDRWENRLEDVFNGR  191
Capsicum annuum (Pepper) PSY1                LRQAALVKRQLRSTD-EL--DVK--------KDIPIPG--TLGLL-SEAYDRCSEVCAEYAKTFYLGTMLMTPERRKAIWAIYVWCRRTDELVDGPNASHITPAALDRWEDRLEDVFSGR  203
Daucus carota (Carrot) PSY1                  LKQAALVREEKRSSRGL-CLDTKRTGSKSFDKSENDDAGMKSWNLLNEAYDRCGEVCAEYAKTFYLGTLLMTPERRRAVWAIYVWCRRTDELVDGPNASHITPKALDRWEKRLNDLFDGQ  181
Eriobotrya japonica (Loquat) PSY2A           LKQAALVEEQS-TVKRK-SLD---------LDERIVTEGLDNWDLLDKAYDRCGEVCAEYAKTFYLGTLLMTPERRRAVWAIYVWCRRTDELVDGPNASYITPKALDRWEKRLTDLFEGR  174
Eriobotrya japonica (Loquat) PSY2B           LKQAALVREPN-TVKKK-SLD---------LDERI-TDGLNNWDLLNKAYDRCGEVCAEYAKTFYLGTLLMTPERRRAVWAIYVWCRRTDELVDGPNASYITPKALDRWEKRLTDLFEGR  173
Medicago truncatula (Barrel clover) PSY2A    LKQAALVKEQRKDIKKR-GLN---------LDDKPIEGDFTNGELLSSAYDRCGDVCAEYAKTFYLGTQLMTQERRKAIWAIYVWCRRTDELVDGPNASHITPKALDRWEQRLTDVFEGR  173
Medicago truncatula (Barrel clover) PSY2B    LKQAGLVREEKRGANIE---F---------DFEKTIEGDFSNGDLLNSAYDRCGEVCAEYAKTFYLGTQLMTQERRKAIWAIYVWCRRTDELVDGPNSSHITPAALDRWEQRLTDVFECR  166
Solanum lycopersicum (Tomato) PSY3           RRVREVVWKQTNVT---N--EY----------LCCKNPRFDP-MFLDEAYELCRKICEEYAKTFYLGTKLMTEERQKAIWAIYVWCRRTDELVDGPNADYMNNSVLDRWEQRLEDIFNNK  166
Cucumis melo (Muskmelon) PSY3                LKVEEIVERQSEGNHFLR--EE----------WCKKKMKFQP-SFLEEAYESCRKICAEYAKTFYLGTLLMTKERQRAIWAIYVWCRRTDELVDGPNAVYMNPKVLDRWEERLEDIFEGR  167
Daucus carota (Carrot) PSY3                  LHINEIVKRQTKRSFAY---------------QSCRKPQFDP-AFLEEAYEQCKNLCSEYAKTFYLGTLLMTKERQKAIWAIYVWCRRTDELVDGPNAAYMSSSVLDRWEERLYDIFNGR  161
Eriobotrya japonica (Loquat) PSY3            LHVQEIVQRQSQTRSVDG--E-----------GSRRRPRFNP-SFLEEAYERCKNLCAEYAKTFYLGTLLMTEERQKAIWAIYVWCRRTDELVDGPNSGYMSSEVLDRWEQRLEDIFEGR  164
Medicago truncatula (Barrel clover) PSY3     LHVQEVVEKQSQTNIFDT--LC----------SSNFKPQFEP-SFLKDAYEMCRNICSEYAKTFYLGTLLMTEERQKAIWAIYVWCRRTDELVDGPNADYMSSAVLDRWEERLHDIFNGC  162
                                              :   :  .                                  :  .**: *  .:* ** *** *** ::::*:***************: ::.   :**** ** :::

Arabidopsis thaliana (Thale cress) PSY        PFDMLDAALADTVARYPVDIQPFRDMIEGMRMDLKKSRYQNFDDLYLYCYYVAGTVGLMSVPVMGIDPKSKATTESVYNAALALGIANQL-TNILRDVGEDARRGRVYLPQDELAQAGLS  322
Eschscholzia californica (Golden poppy) PSY1 AYDMLDAALSDTVARFPVDIQPFRDMIEGMRMDLRKSRYKNFDELYLYCYYVAGTVGLMSVPVMGIEPESQATTESIYSAALALGIANQL-TNILRDVGEDARRGRVYLPQDELAQAGLS  325
Papaver somniferum (Opium poppy) PSY2        PYDMLDAALSDTVQKFPVDIQPFKDMIEGMRLDLKKSRYQNFDELYLYCYYVAGTVGLMSVPVMGIEPESKATTESVYGAALALGIANQL-TNILRDVGEDARRGRVYLPQDELMQSGLS  325
Medicago truncatula (Barrel clover) PSY1     PFDMLDAALSDTVNRFPVDIQPFKDMIEGMRMDLKKSRYKSFDELYLYCYYVAGTVGLMSVPVMGISTHSQATTESVYNAALALGIANQL-TNILRDVGEDARRGRVYLPQDELTLAGLS  333
Cucumis melo (Muskmelon) PSY1                PFDMLDAALADTVTKFPVDIQPFKDMIEGMRMDLRKSRYKNFDELYLYCYYVAGTVGLMSVPVMGIAPESQASTESVYNAALALGIANQAPPNILRDVGEDARRGRIYLPQDELAQAGLS  327
Eriobotrya japonica (Loquat) PSY1            PFDMLDAALSDTVTKFPVDIQPFKDMIEGMRMDLRKSRYQSFDELYLYCYYVAGTVGLMSVPVMGISPESQATTESVYNAALALGIANQL-TNILRDVGEDARRGRIYLPQDELAEAGLS  321
Manihot esculenta (Cassava) PSY1             PFDMLDAALSDTVTKFPVDIQPFKDMIEGMRMDLKKSRYNNFDELYLYCYYVAGTVGLMSVPVMGIAPESQASTESVYNAALALGIANQL-TNILRDVGEDARRGRIYLPQDELAQAGLS  327
Manihot esculenta (Cassava) PSY2             PFDMLDAALSDTVTKFPVDIQPFKDMIEGMRMDLKKSRYKNFDELYLYCYYVAGTVGLMSVPVMGIAPESQASTESVYNAALALGIANQL-TNILRDVGEDARRGRIYLPQDELAQAGLS  325
Daucus carota (Carrot) PSY2                  PFDMLDAALSDTVMKFPVDIQPFKDMIEGMRMDLKKSRYKNFDELYLYCYYVAGTVGLMSVPIMGIAPNSQATTESVYNAALALGLANQL-TNILRDVGEDARRGRVYLPQDELAQAGLS  334
Solanum lycopersicum (Tomato) PSY2           PFDMLDAALSDTVSRFPVDIQPFRDMVEGMRMDLWKSRYNNFDELYLYCYYVAGTVGLMSVPIMGIAPESKATTESVYNAALALGIANQL-TNILRDVGEDARRGRVYLPQDELAQAGLS  334
Solanum lycopersicum (Tomato) PSY1           PFDMLDGALSDTVSNFPVDIQPFRDMIEGMRMDLRKSRYKNFDELYLYCYYVAGTVGLMSVPIMGIAPESKATTESVYNAALALGIANQL-TNILRDVGEDARRGRVYLPQDELAQAGLS  310
Capsicum annuum (Pepper) PSY1                PFDMLDAALSDTVSKFPVDIQPFRDMIEGMRMDLRKSRYRNFDELYLYCYYVAGTVGLMSVPIMGIAPESKATTESVYNAALALGIANQL-TNILRDVGEDARRGRVYLPQDELAQAGLS  322
Daucus carota (Carrot) PSY1                  PYDMYDAALADTVSTYPVDIQPFKDMIDGMRMDLKKSRYQTFDELYLYCYYVAGTVGLMSVPVMGIAPESKATTESVYSAALALGIANQL-TNILRDVGEDARRGRIYLPQEELKLAGIT  300
Eriobotrya japonica (Loquat) PSY2a           PYDMYDAALSDTVTKYPVDIQPFRDMVEGMRLDLRKSRYQNFDELYLYCYYVAGTVGLMSVPVMGICPESRASTESVYNAALALGIANQL-TNILRDVGEDARRGRVYLPQDELAQAGLS  293
Eriobotrya japonica (Loquat) PSY2b           PYDMYDAALSDTVAKYPVDIQPFRDMVEGMRLDLRKSRYQNFDELYLYCYYVAGTVGLMSVPVMGISPESKASTESVYNAALALGIANQL-TNIPRDVGEDARRGRVYLPQDELAQAGLS  292
Medicago truncatula (Barrel clover) PSY2A    PYDMYDAALSDTVTKYPVDIQPFKDMIEGMRLDLRKSRYNNFDELYLYCYYVAGTVGLMSVPVMGIEPQSKASTESIYNAALALGIANQL-TNILRDVGEDARRGRVYLPQDELAQAGLS  292
Medicago truncatula (Barrel clover) PSY2B    PYDMFDAALSLSVSKYPVDIQPFKDMIEGMRMDLTKSRYNNFDELYLYCYYVAGTVGLMSVPVMGIAPESKASTESIYNAALALGIANQL-TNILRDVGEDARRGRVYLPQDELAQAGIS  285
Solanum lycopersicum (Tomato) PSY3           PYDMLDAALTDTICKFPLDIKPFKDMIDGMRMDTRKSRYANFQELYMYCYCVAGTVGLMSVPIMGIAPESCVSAQTVYNAALHLGIGNQL-TNILRDVGEDALRGRVYLPQDELAQFGIR  285
Cucumis melo (Muskmelon) PSY3                PYDLLDAALSHTVSKFPIDMKPFRDMIEGMRMDTKKCRYENFEELYLYCYYVAGTVGLMSVPVMGIAPDSSLSTHTIYSAALHLGIGNQL-TNILRDVGEDATRGRVYLPQDELAQFGLC  286
Daucus carota (Carrot) PSY3                  PFDMLDAALSDTVQNFPLDIKPFGDMIEGMRMDTRKTRYKNFQEIYLYCYYVAGTVGLMSVPVMGIPPESTVPAQSIYDAALYLGIGNQL-TNILRDVGEDTLRGRIYLPQDELEQFGIT  280
Eriobotrya japonica (Loquat) PSY3            PYDILDAALTNTVSNFPLDIKPFRDMIEGMRMDTQKCRYQNFQELYLYCYYMAGTVGLMSVPVMGIAPDSRISAQSTYDSALYLGIGNQL-TNILRDVGEDAMRGRVYLPQDELARFGLC  283
Medicago truncatula (Barrel clover) PSY3     PYDMLDAALTDTISKFPLDIKPFRDMIEGMRMDTRKSRYKNFEELYLYCYYVAGTVGLMSVPVMGIAPESLIPAQSVYKSALYLGIGNQL-TNILRDVGEDALRGRVYLPQDELGEFGLC  281
                                               :*: *.**: ::  :*:*::** **::***:*  * ** .*:::*:*** :**********:***  .*   :.: * :** **:.**   ** ******: ***:****:**   *:

Arabidopsis thaliana (Thale cress) PSY        DEDIFAGKVTDKWRNFMKMQLKRARMFFDEAEKGVTELSAASRWPVWASLLLYRRILDEIEANDYNNFTKRAYVGKVKKIAALPLAYAKSVLKTSSSRLSI------   423
Eschscholzia californica (Golden poppy) PSY1 DEDIFAGKVTDKWRSFMKKQIQRARMFFDEAEKGVTQLSSASRWPVWASLLLYRQILDEIEANDYNNFTKRAYVSKVKKIVALPVAYARSIVGVSRSSPLVKT----   428
Papaver somniferum (Opium poppy) PSY2        DEDIFAGKVTDKWRSFMKKQIKRARMFFDEAESGVTQLSASSRWPVWASLLLYRQILDEIEANDYNNFTKRAYVSKAKKVVALPVAYAKSILGSTKKTPQTLQASKK   432
Medicago truncatula (Barrel clover) PSY1     DDDIFAGKVTDKWRNFMKSQIKRARTFFDEAEKGVTELNEQSRWPVWASLLLYRQILDEIEANDYNNFTKRAYVSKTKKLLSLPLAYARSMVPPSKKLSHV------   434
Cucumis melo (Muskmelon) PSY1                DEDIFAGRVTDKWRNFMKNQIKRARMFFDEAEKGVLELNKASRWPVWASLLLYRQILDEIEANDYDNFTKRAYVSKAKKILALPMAYGRALLGPS------------   422
Eriobotrya japonica (Loquat) PSY1            DADIYAGKVTDKWRSFMKDQIKRARMFFDEAEKGVTELSEASRWPVLASLLLYRQILDEIEANDYNNFTRRAYVSKAKKLLALPIAYTKSIIRPPRTSPELRKYNL-   427
Manihot esculenta (Cassava) PSY1             DEDIFAGEVTNKWRNFMKNQIKRARMFFNEAEKGVTELSAASRWPVWASLLLYKQILDEIEANDYNNFTERAYVNKAKKLAFLPIAYARSFVGSSRVSPPLANP---   431
Manihot esculenta (Cassava) PSY2             DDDIFAGKVTDKWRNFMKNQIKRARMFFNEAEKGVTELSAASRWPVWASLLLYRRILDEIEANDYNNFTKRAYVSKTKKIASLPIAYARSFVGPSRMSSPVTKA---   429
Daucus carota (Carrot) PSY2                  DEDIFAGKVTDKWRNFMKKQIKRARMFFDEAQIGVRELSPASRWPVWASLLLYRQILDEIEANDYNNFTKRAYVSKPKKILALPVAYAKAFAPTARTSSTFLKT---   438
Solanum lycopersicum (Tomato) PSY2           DEDIFAGKVTDKWRIFMKKQIQRARKFFDEAEKGVTELSSASRWPVLASLLLYRKILDEIEANDYNNFTRRAYVSKPKKLLTLPIAYARSLVPPKSTSSPLAKT---   438
Solanum lycopersicum (Tomato) PSY1           DEDIFAGRVTDKWRIFMKKQIHRARKFFDEAEKGVTELSSASRFPVWASLVLYRKILDEIEANDYNNFTKRAYVSKSKKLIALPIAYAKSLVPPTKTASLQR-----   412
Capsicum annuum (Pepper) PSY1                DEDIFAGRVTDKWRIFMKKQIQRARKFFDEAEKGVTELSAASRWPVLASLLLYRRILDEIEANDYNNFTKRAYVSKPKKLIALPIAYAKSLVPSTRT----------   419
Daucus carota (Carrot) PSY1                  PEYIFKGKVTDKWRSFMKGQIKRARMFFDEAEKGVAELSSASRWPVWASLLLYKQILDAIEANDYDNFTKRAYVGKAKKLVSLPLAYSRALFAPSTVR---------   398
Eriobotrya japonica (Loquat) PSY2a           DDDIFRGKVTDKWQSFMKGQIQRARMFFDEAEKGVSELNSASRWPVWASLLLYRQILDVIEANGYDNFTKRAYVGKAKKFVSLPVAYGRAIIGPSKLTKQLVPR---   397
Eriobotrya japonica (Loquat) PSY2b           DNDIFRGKVTDKWQSFMKGQIKRARMFFDEAEKGVSELNSASRWPVWASLLLYRQILDAIEANGYDNFTKRAYVGKAKKLASLPVAYGRAILGPSKLTKQLVPR---   396
Medicago truncatula (Barrel clover) PSY2A    DDDIFRGRVTDKWRNFMKGQIKRARMFFDEAEKGVSELSSASRWPVWASLLLYRQILDSIEANDYNNFTKRAYVGKAKKLLSLPVAFGIATFGPQKLAKMTTR----   395
Medicago truncatula (Barrel clover) PSY2B    DEDIMRGKVTDKWRNFMKGQIKRARMFFDEAEKGVSELSSASRWPVWAALLLYRQILDSIEANDYNNFTKRAYVGKVNKLLSLPAAYGVALLGPKKLTKLFMR----   388
Solanum lycopersicum (Tomato) PSY3           DEDVFARKVTDQWREFMKEQIRRARFYFNLAEEGASHLNKASRWPVWSSLILYMKILDAIEENDYDNLTKRAYVGRAKKLVTLPVSYARALSLPSLAIQ--------   384
Cucumis melo (Muskmelon) PSY3                DADILRMRVSDKWREFMKEQIKRARFYFKLAEEGASQLDKASRWPVWSSLMLYRKILEAIEENDYNNFTKRAYVGRSKKLLALPLAYTKSISPPTLVFN--------   385
Daucus carota (Carrot) PSY3                  EEEVFSRKVSDKWREFMKMQITRARYYFNQAEEGASQLDKDSRWPVWSSLMLYREILDAIEDNDFDNLTKRAYVGRTKKLLMLPLAYTRSLSISHFMSGRTNVML--   385
Eriobotrya japonica (Loquat) PSY3            DNDVFSRKVTDKWRAFMKEQITRARFCFNLAEEGASQLDKASRWPVWSSLLIYRNILDAIEDNDYDNLTKRAYVQRAKKLLMLPLAYTRSLSTHNLMSQ--------   382
Medicago truncatula (Barrel clover) PSY3     DNDVFSRKVSERWREFMKQQIARARFYFNSAEEGASHLDKASRWPVWSSLILYRKILDAIEDNDYDNLTKRAYVGRTEKFVSLPAAYTRSLSIPKTESHASFTRHM-   387
                                                 :   .*:::*: *** *: ***  *. *: *. .*.  **:** ::*::* .**: ** *.::*:*.**** : :*.  ** ::  :
```

Figure S4

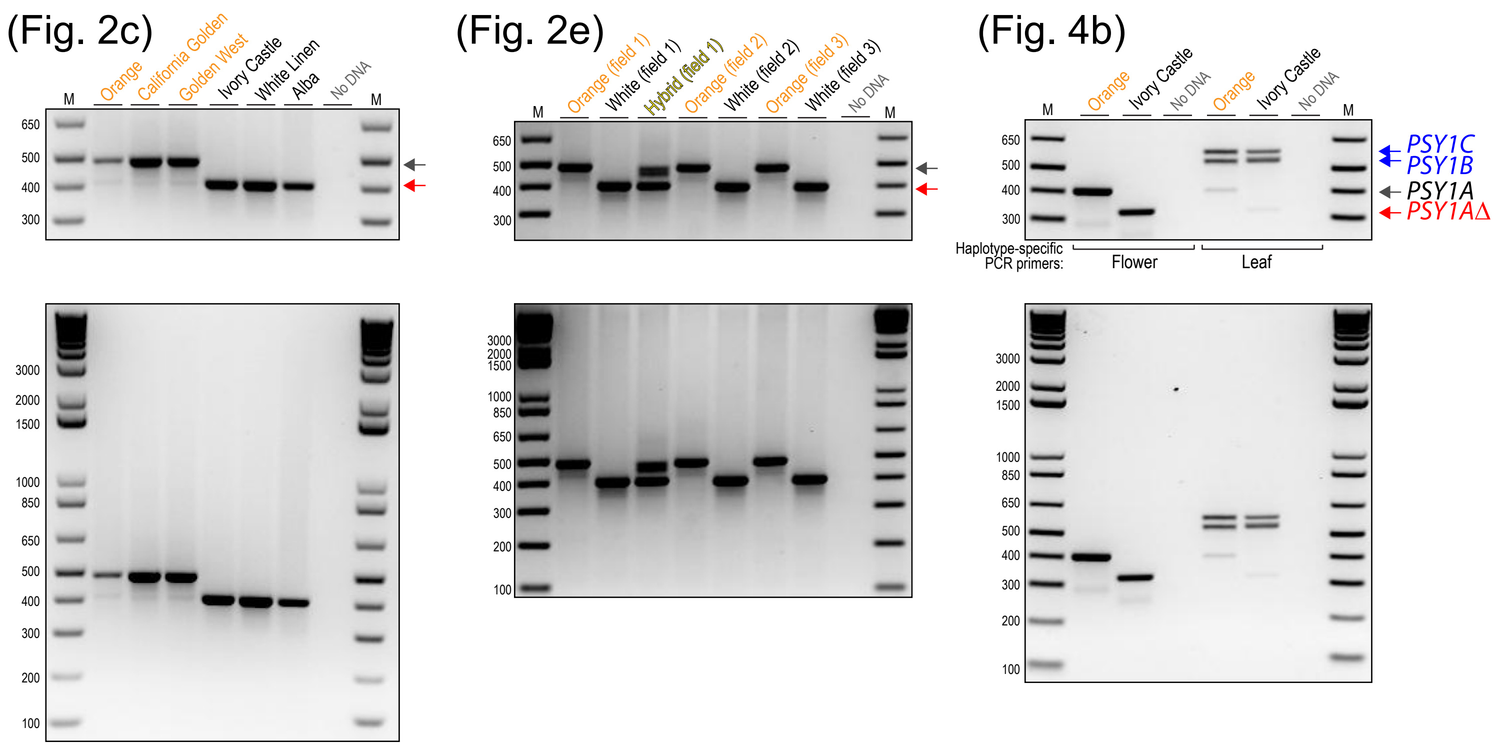

(Fig. 2c)
M
Orange
California Golden
Golden West
Ivory Castle
White Linen
Alba
No DNA
M
650
500
400
300
3000
2000
1500
1000
850
650
500
400
300
200
100
(Fig. 2e)
M
Orange (field 1)
White (field 1)
Hybrid (field 1)
Orange (field 2)
White (field 2)
Orange (field 3)
White (field 3)
No DNA
M
650
500
400
300
3000
2000
1500
1000
850
650
500
400
300
200
100
(Fig. 4b)
M
Orange
Ivory Castle
No DNA
Orange
Ivory Castle
No DNA
M
650
500
400
300
PSY1C
PSY1B
PSY1A
PSY1AΔ
Haplotype-specific
PCR primers:
Flower
Leaf
3000
2000
1500
1000
850
650
500
400
300
200
100
